# Biochemical, Antioxidant Properties and Antimicrobial Activity of Epiphytic Leafy Liverwort *Frullania dilatata* (L.) Dumort

**DOI:** 10.3390/plants12091877

**Published:** 2023-05-04

**Authors:** Ozcan Simsek, Kerem Canli, Atakan Benek, Dilay Turu, Ergin Murat Altuner

**Affiliations:** 1 Department of Forestry, Yenice Vocational School, Çanakkale Onsekiz Mart University, Çanakkale 17950, Türkiye; ozcansimsek@comu.edu.tr; 2 Department of Biology, Faculty of Science, Dokuz Eylül University, Izmir 35390, Türkiye; 3 Fauna and Flora Research and Application Center, Dokuz Eylül University, Izmir 35390, Türkiye; 4 Department of Biology, Graduate School of Natural and Applied Sciences, Kastamonu University, Kastamonu 37150, Türkiye; atakan.benek@hotmail.com; 5 Department of Biology, Graduate School of Natural and Applied Science, Dokuz Eylül University, Izmir 35390, Türkiye; dilayturu@gmail.com; 6 Department of Biology, Faculty of Science, Kastamonu University, Kastamonu 37150, Türkiye; ergin.murat.altuner@gmail.com

**Keywords:** antimicrobial activity, antioxidant properties, bioactive compounds, *Frullania dilatata*, liverworts

## Abstract

In this study, the biochemical, antioxidant properties, and antimicrobial activity of the epiphytic leafy liverwort *Frullania dilatata* (L.) Dumort were investigated. Due to the scarcity and difficulty in obtaining liverworts, research on their bioactivity is limited; thus, this study aimed to uncover the potential of *F. dilatata*. The antimicrobial activity was evaluated against various microorganisms, including food isolates, clinical isolates, multidrug-resistant strains, and standard strains, using the disk diffusion method and determining the minimum inhibitory concentration (MIC) values. This study represents the first antioxidant investigation on *F. dilatata* and an antimicrobial study using ethanol extract and the disk diffusion method. Notably, susceptibility was observed in *Enterococcus faecalis* ATCC 29212, *Enterococcus faecium* FI, *Listeria monocytogenes* ATCC 7644, *Providencia rustigianii* MDR, and *Staphylococcus aureus* ATCC 25923. The antioxidant capacity was assessed using the DPPH method, emphasizing the high scavenging performance. Gas chromatography-mass spectrometry (GC-MS) analysis identified the primary compounds as frullanolide (19.08%), 2,3-Dimethylanisole (15.21%), linoleic acid (11.11%), palmitic acid (9.83%), and valerenic acid (5.3%). The results demonstrated the significant antimicrobial activity of *F. dilatata* against the tested microorganisms and its potent antioxidant properties. These findings emphasize the potential of *F. dilatata* as a promising source of natural antimicrobial and antioxidant agents, underscoring the importance of further investigation into its bioactive compounds and elucidating the mechanisms of action in future studies.

## 1. Introduction

Liverworts (Marchantiophyta) are a group of non-vascular plants that comprise over 9000 species, representing an important component of the bryophyte division [1]. These plants have a simple structure and possess a wide range of secondary metabolites, including terpenoids, phenolic compounds, and flavonoids, which contribute to their diverse biological activities [2]. Although liverworts are not generally included in mammalian diets, including those of humans, mainly due to their unpalatability and potential allergenic effects, they have been traditionally used in ethnomedicine for treating various ailments such as wounds, digestive disorders, and respiratory issues [3]. Recent scientific investigations have also revealed the potential of liverworts as a source of biologically active compounds with antimicrobial, antioxidant, anti-inflammatory, and anticancer properties. As a result, liverworts have attracted increasing attention from researchers who seek to explore their potential applications in modern medicine and pharmacology [4].

The chemical composition of liverworts contains unique compounds not found in other plants, including bis-bibenzyls, bibenzyls, and terpenoids. These compounds exhibit various biological activities such as antimicrobial, antifungal, cytotoxic, and neuroprotective properties [5,6]. Notably, some bibenzyls have been shown to be effective in the treatment of neurodegenerative disorders such as Alzheimer’s and Parkinson’s diseases [7]. Liverworts also possess oil bodies, which are specialized organelles containing lipids, terpenoids, and other metabolites. These oil bodies serve as storage sites and are involved in stress response and defense mechanisms against herbivory [8].

Significant antimicrobial activity against Gram-positive and Gram-negative bacteria was observed in extracts from the liverwort species *Porella arboris-vitae* [9]. Additionally, *Marchantia polymorpha* was found to have strong antioxidant properties due to its high phenolic content [10]. In another study, the liverwort *M. polymorpha* demonstrated its potential as an anticancer agent, as its extract exhibited cytotoxic effects on human cancer cell lines [11]. These studies underscore the importance of continued investigation into the bioactivities of liverworts. However, the scarcity of liverworts and the limited amount of substances obtained during the extraction process contribute to the bioactivity studies of liverworts being far more restricted compared to other plant groups. Despite the limitations of the studies, it is crucial to contribute new information to the literature and evaluate the growing body of knowledge collectively over time.

Free radicals exert detrimental effects on living organisms, and antioxidants play a crucial role in neutralizing these radicals within biological cells. Plant compounds with antioxidant properties, such as vitamins, unsaturated fatty acids, enzymes, anthocyanins, carotenoids, flavonoids, phenolic acid derivatives, and cofactors, have the capacity to capture free radicals. This has increased interest in utilizing these plant metabolites in phytotherapy [12].

Antimicrobial agents are vital for diminishing the impact of infectious diseases worldwide. However, the emergence of multidrug resistance in pathogenic bacteria has reduced or even eliminated the effectiveness of commonly used antimicrobials, resulting in a significant public health threat [13,14]. The key to combating antibiotic resistance, a serious public health issue, lies in the discovery of novel compounds [15].

In particular, remote and economically disadvantaged areas often struggle to obtain essential antimicrobial agents, exacerbating the problem. In response to these challenges, the World Health Organization (WHO) has highlighted the importance of exploring alternative sources for novel antimicrobial compounds. Plants, with their vast array of bioactive constituents, are recognized by the WHO as a promising source for the discovery of new antimicrobial agents to combat the growing threat of antibiotic resistance and address the healthcare needs of vulnerable populations [16].

*Frullania* is one of the largest and taxonomically most complex genera of leafy liverworts. The genus *Frullania* comprises approximately 400 species, according to the literature [17]. *F. dilatata*, a medium-sized, dorsiventrally flattened, leafy liverwort, often grows as an epiphyte in close proximity to the substrate. Its color ranges from green to reddish-black, contingent on age and growing conditions. *F. dilatata* can be easily recognized due to its large and warty perianths [18].

In this study, the antimicrobial, antioxidant, and biochemical composition of *F. dilatata* ethanol extracts were investigated. There has been only one previous study on *F. dilatata*, which examined the antimicrobial activity of water extracts against *Staphylococcus aureus*. However, they could not obtain clear results with the agar well method and therefore used the MIC technique. In contrast, our study investigates the antimicrobial activity against a wide range of strains, setting it apart from the limited previous research [19]. Our study also stands out as the first one to obtain results on the antimicrobial potential of ethanol extracts using the disk diffusion method. Furthermore, our research is the first to investigate the antioxidant properties and biochemical composition of *F. dilatata*.

## 2. Results

The antimicrobial activity of the ethanol extract from *F. dilatata* was assessed against 13 bacterial and 2 yeast strains using the disk diffusion method. The inhibition zones formed around the extract-loaded disks are presented in Table 1. Gentamicin was used as a positive control, and its results are also included in Table 1. Disks impregnated with 200 µL of absolute ethanol, without the extract, served as the negative controls. The ethanol was allowed to evaporate under the same conditions and duration as in the experimental procedure, resulting in no observed inhibition zones. An ANOVA test was performed to determine the significance of differences between the parallels, yielding a *p*-value of 0.9625. As the *p*-value is greater than 0.05, it can be concluded that there is no significant difference between the parallels, indicating that the experimental results are reliable and consistent.

The minimum inhibitory concentration (MIC) values for the ethanol and water extracts of *F. dilatata* were determined against 13 bacterial and 2 yeast strains, as presented in Table 2. MIC values for the ethanol extract ranged from 0.1285 to 1.028 mg/mL, while the water extract displayed MIC values of 21.44 mg/mL against two of the tested microorganisms. No inhibitory effect was observed for several microorganisms in both the ethanol and water extracts. As all the parallels yielded the same results, the ANOVA test was deemed unnecessary, and the *p*-value is considered to be 1.

The antioxidant activity of the *F. dilatata* ethanol extract was assessed using the DPPH (2,2-diphenyl-1-picrylhydrazyl) method. The analysis revealed that the extract exhibited concentration-dependent antioxidant activity. Ascorbic acid was used as a positive control and for comparison purposes. For the negative controls, ethanol without the extract was used. The results of the antioxidant activity are presented in Table 3 below. The DPPH radical scavenging assay revealed that the EC_50_ value for *F. dilatata* was 0.03169 mg/mL. An ANOVA test was performed to determine the significance of differences between the parallels, yielding a *p*-value of 0.997492. As the *p*-value is greater than 0.05, it can be concluded that there is no significant difference between the parallels, indicating that the experimental results are reliable and consistent. Furthermore, the Pearson correlation coefficient was calculated to be 0.8690298, indicating a very strong positive correlation between the concentration of the extract and the DPPH scavenging activity. An additional ANOVA test was conducted to compare the difference in DPPH activity between the extract and ascorbic acid, resulting in a *p*-value of 0.1754. This indicates that the difference between the effects of ascorbic acid and the extract on DPPH activity is statistically insignificant.

The chemical content of *F. dilatata*’s ethanol extract was analyzed via GC/MS. Chemicals constituting more than 0.5% were considered the main components and are listed in Table 4, where each compound’s chemical structure, chemical formula, molecular weight, and known activities are provided in the respective columns.

## 3. Discussion

The antimicrobial activity study for *F. dilatata* was previously performed only by Nikolajeva et al. [19]. In the study conducted by Nikolajeva et al., the antimicrobial activity of the water extract of *F. dilatata* against the *S. aureus* strain was determined using the minimum inhibitory concentration method (MIC). In our study, we also performed the MIC assay to evaluate the antimicrobial activity of both the ethanol and water extracts of *F. dilatata* against *S. aureus*. We obtained consistent results with this previous research when examining the single strain of *S. aureus*. Our MIC results showed that the ethanol extract had an effective concentration of 0.257 mg/mL against *S. aureus* ATCC25923, while the water extract had an effective concentration of 21.44 mg/mL against the same strain. These findings revealed that the ethanol extract was much more potent and effective at significantly lower concentrations compared to the water extract, which is consistent with the results obtained for the water extract in the previous study [19]. In our investigation, the largest inhibition zone of 11 mm was detected against the *S. aureus* ATCC 25923 strain using the disk diffusion method for the ethanol extract. *S. aureus* is found in the human flora, primarily located in the nose and skin of most healthy individuals, and is an important human pathogen that causes various clinical manifestations. This bacterium is a significant cause of hospital-acquired infections, leading to complications such as surgical site infections, bloodstream infections, and pneumonia. Additionally, *S. aureus* is associated with a wide range of health issues, including skin and soft tissue infections, food poisoning, and more severe cases such as endocarditis and sepsis. The increasing prevalence of antibiotic-resistant strains, such as methicillin-resistant *S. aureus* (MRSA), highlights the urgent need for effective prevention and control strategies to mitigate the impact of this pathogen on public health [31]. Given the significant impact of *S. aureus* on public health and the increasing prevalence of antibiotic-resistant strains, our findings demonstrating the antimicrobial activity of *F. dilatata* against the *S. aureus* ATCC 25923 strain highlight the potential of this liverwort as a valuable source for developing alternative therapeutic agents.

There is a significant increase in bacterial drug resistance worldwide. By 2050, infectious diseases caused by MDR bacteria are expected to cause more deaths than cancer [32]. *P. rustigianii* is an invasive enteric MDR pathogen that can colonize the human intestinal tract, be isolated from human feces, and cause diarrhea [33]. In our study, using the disk diffusion method for the ethanol extract, we observed a 9 mm activity against *P. rustigianii*, and according to the MIC results for the ethanol extract, the effective concentration was 1.028 mg/mL. The limited bioactivity research on this bacterium means we still have much to learn about its properties. On the other hand, finding an effect against an MDR strain is of considerable importance. As research progresses, it will be crucial to understand the mechanism of efficacy against not only *P. rustigianii* but also other MDR bacteria in the future.

*Listeria monocytogenes* is a foodborne bacterium with significant pathogenic activity. It causes serious diseases, including listeriosis, characterized by symptoms such as fever, muscle aches, and gastrointestinal distress. In more severe cases, listeriosis can lead to meningitis, septicemia, and even death, particularly among vulnerable populations such as pregnant women, newborns, the elderly, and immunocompromised individuals [34,35]. The discovery of new alternative compounds against listeriosis becomes critical for the food industry, which is of great importance globally, as the incidence of listeriosis continues to rise and antibiotic resistance becomes more prevalent. The disk diffusion assay conducted in this research demonstrated a 9 mm inhibition zone against *L. monocytogenes*, indicating the potential of *F. dilatata* to serve as a source of new antimicrobial agents. Additionally, the MIC results revealed an effective concentration of 0.257 mg/mL for the ethanol extract and 21.44 mg/mL for the water extract against *L. monocytogenes*. These results are particularly promising considering the urgent need for novel approaches to combat *L. monocytogenes* and the increasing challenge of antibiotic resistance. Further research is necessary to elucidate the specific compounds responsible for this activity and to investigate their mechanisms of action, as well as to evaluate their potential for development into effective therapeutic agents against listeriosis and other related diseases.

Our study revealed that *F. dilatata* demonstrated considerable antimicrobial effects against both *E. faecalis* ATCC 29212 and *E. faecium* FI, showing zone diameters of 9 mm and 8 mm, respectively, in the disk diffusion assay for the ethanol extract. The MIC results for the ethanol extract against these strains were 0.1285 mg/mL for *E. faecalis* ATCC 29212 and 1.028 mg/mL for *E. faecium* FI. The impact on *E. faecium* FI is of particular importance, given the role of *E. faecium* in infections associated with healthcare settings. According to the World Health Organization (WHO), such infections affect a significant proportion of hospitalized patients in high-income countries, leading to increased morbidity and mortality rates [36]. Both *E. faecalis* and *E. faecium* are recognized as leading causes of infections in healthcare environments, with vancomycin-resistant *E. faecium* (VREfm) posing a serious threat to immunocompromised patients [37]. The growing prevalence of vancomycin resistance among *E. faecium* clinical isolates highlights the urgent need for alternative therapeutic options. The observed antimicrobial activity of *F. dilatata* against both *E. faecalis* ATCC 29212 and *E. faecium* FI in our study could potentially contribute to the discovery of new treatment strategies for tackling infections caused by these pathogens in healthcare settings.

In our investigation, we assessed the antifungal activity against two yeast strains, *C. albicans* DSMZ 1386 and *C. tropicalis* CI, using the MIC method for the ethanol extract. We obtained MIC results of 0.257 mg/mL for *C. albicans* DSMZ 1386 and 0.514 mg/mL for *C. tropicalis* CI. While no inhibitory effects were observed in the disk diffusion assay, the presence of antifungal activity in the MIC results might be attributed to the hydrophobic nature of the antifungal active compounds present in the extract. This hydrophobicity could potentially hinder diffusion in the agar medium or cause the compounds to evaporate before diffusing from the disk due to their high volatility.

Although the antimicrobial activity observed in the disk diffusion assay was not found to be extremely high, the fact that the plant studied is a liverwort. It, therefore, has a limited amount of plant material available for extraction, making the observed effect quite significant, even if it appears to be low. In contrast, the MIC results for the ethanol extract showed potent antimicrobial activity at relatively low concentrations against various strains, including yeasts. Moreover, the MIC results for the ethanol extract were consistently more effective at lower concentrations compared to the water extract for many strains.

This study is the first to reveal the antioxidant activity of *F. dilatata*. As a result of the antioxidant activity study performed using the DPPH method, it was determined that the ethanol extract of *F. dilatata* exhibited a remarkable effect of 89.294% at a concentration of 200 µg/mL. This result is notably close to the 94.515% effect of ascorbic acid, a well-known antioxidant agent, at the same concentration. The strong antioxidant properties exhibited by *F. dilatata*, nearly rivaling those of ascorbic acid, highlight its potential as a valuable natural source of antioxidants.

*F. dilatata* extract, which has been analyzed using gas chromatography-mass spectrometry (GC-MS), has been found to contain beta-Elemene and linoleic acid as its major components. Previous studies have reported that both of these compounds possess notable antioxidant properties [20,30]. Antioxidants are essential in the body’s defense against oxidative stress, which is responsible for the damage caused by free radicals. The presence of these compounds in *F. dilatata* suggests that the extract has potential therapeutic value in combating oxidative stress-related diseases.

The beta-Elemene, a sesquiterpene compound, has been previously associated with various biological activities, including anti-inflammatory, antitumor, and immunomodulatory effects [20]. Its antioxidant properties contribute to its overall therapeutic potential, and its presence in *F. dilatata* extract may be responsible for a portion of the observed antioxidant activity.

Similarly, Linoleic acid, an essential polyunsaturated fatty acid, has been known to play a crucial role in various physiological processes, including cellular signaling, inflammation regulation, and membrane structure maintenance [30]. Its antioxidant properties help protect cellular components from oxidative damage, which could, in turn, help maintain overall cellular health and function.

The findings from this study suggest that the antioxidant activity observed in *F. dilatata* extract can be attributed, at least in part, to the presence of beta-Elemene and linoleic acid. These compounds may work synergistically to provide potent antioxidant effects, making *F. dilatata* a potentially valuable natural resource for the development of new antioxidant agents or supplements. Further research is necessary to elucidate the precise mechanisms through which these compounds exert their antioxidant effects and to explore the full range of potential applications of *F. dilatata* extract in promoting health and well-being.

*Frullania* species are notable as liverworts that cause very intense allergenic contact dermatitis. *F. dilatata* used in this study is one of the species that causes allergenic contact dermatitis. As a result of GC-MS analysis of *F. dilatata* extract, it was determined that it contains Frullanolide in its structure. This substance is a sesquiterpene lactone and is known to cause allergenic contact dermatitis [38]. As a result of the study conducted by Chimplee et al. [24], it was determined that the same substance showed anti-breast cancer activity.

The antimicrobial and antioxidant activity results of *F. dilatata* are consistent with the compounds identified through GC/MS analysis and the known bioactivity properties of these compounds. However, it is also possible that the activity may be influenced by unidentified compounds not found in the universal libraries. Further studies should investigate the identification and activity of these unknown compounds, particularly in terms of their synergistic and antagonistic effects on bioactivity.

## 4. Materials and Methods

### 4.1. Plant Sample

The liverwort *F. dilatata* utilized in this study was collected from Canakkale Kazdaglari, specifically in the Yenice section of the Kazdağları Mountains at the coordinates 39°55′50.80″ N, 27°12′42.60″ E, and an altitude of 356 m. The collection took place on 22 June 2021 within a 30-m diameter area. The substrate in the area consisted of *Pinus nigra* subsp. *pallasiana* (Lamb.) Holmboe (Anatolian black pine) contributed to the formation of a microecosystem that allowed for the dense growth of the species due to the high moisture content in the region. Sustainable collection practices were employed, ensuring the continuity of the population without causing any significant damage to the natural habitat.

During the collection process, care was taken to obtain samples from the same substrate at the same time. After collection, the liverwort samples were dried in a shaded area, away from direct sunlight, and then stored in a dark and cool environment to preserve their properties until they were used in the experiments. The liverwort samples were identified by Dr. Özcan Simsek, and voucher specimens have been preserved at the Dokuz Eylul University’s Research and Application Center for Fauna Flora (FAMER).

This study was conducted under the supervision and with the permission of the Turkish Ministry of Agriculture and Forestry, General Directorate of Nature Conservation and National Parks (permit number E-21264211-288.04-3471957).

### 4.2. Extraction Process

The extraction method for both ethanol and water extracts involved the following steps: After the drying process, the plant samples were ground to a fine powder using a grinder. For the ethanolic extract, 10 g of powdered sample were mixed with 200 mL of absolute ethyl alcohol (Sigma-Aldrich, Saint Louis, MO, USA), while for the water extract, 5 g of powdered sample were combined with 100 mL of ultra-pure water. Both samples were then shaken at 140 rpm at room temperature for 96 h, instead of the standard 48 or 72 h typically preferred for seed plants, to obtain a higher yield of compounds. After shaking, both the ethanolic and water extracts were filtered through Whatman No. 1 filter paper, primarily for the removal of solid residues. The filtrates were collected in evaporation flasks.

For the ethanolic extract, the extraction solvent was evaporated under a vacuum using a rotary evaporator (Buchi R3, BÜCHI, Labortechnik AG, Postfach, Flawil, Switzerland) at 30 °C. The dried crude ethanolic extract was collected using an analytical balance for accurate weighing and used to prepare the extracted stock, yielding 0.09 g in 35 mL (2.57 mg/mL). The ethanolic extract stock was stored at +4 °C in a refrigerator until the time of application. For the MIC test, the dried crude ethanolic extract was dissolved in DMSO at a non-toxic concentration of 1%. In addition, a 1% DMSO solution was used as a negative control to ensure no toxic effect on the microorganisms during the experiment.

The water extract was dried using a HyperCOOL HC3110 lyophilizer (Gyrozen, Gimpo, Korea). The water extract yielded 0.536 g in 10 mL (53.6 mg/mL), and the final stock was prepared and passed through a 0.45 µm Minipore membrane filter to ensure sterility. The water extract stock was stored at −35 °C in a deep freezer until the time of application [15].

### 4.3. Microorganisms

In this study, we used 8 standard, 1 clinical isolate, 3 multidrug resistant, and 1 food-isolated bacteria, as well as 1 standard and 1 clinical isolated yeast strain. The standard gram-positive bacteria included *E. faecalis* ATCC 29212, *L. monocytogenes* ATCC 7644, *S. aureus* ATCC 25923, and *S. hominis* ATCC 27844. The standard gram-negative bacteria comprised *E. coli* ATCC 25922, *P. aeruginosa* DSMZ 50071, *S. typhimurium* SL1344, and *A. baumannii* CECT 9111. The gram-negative clinically isolated bacterium was *K. pneumoniae*. The gram-positive multidrug-resistant strain was *S. pneumoniae*, while the gram-negative multidrug-resistant strains included *P. rustigianii* and *A. baumannii*. The gram-positive food-isolated bacterium was *E. faecium*. The standard yeast was *C. albicans* DSMZ 1386, and the clinically isolated yeast was *C. tropicalis*. The strains were obtained from the Department of Biology at Dokuz Eylül University’s Faculty of Science (İzmir, Türkiye).

### 4.4. Microorganism Inoculum Preparation

Bacterial strains used to determine the antimicrobial activity of *F. dilatata* ethanol extract were incubated at 37 °C for 24 h, while yeast strains were incubated at 27 °C for 48 h. The inocula of bacterial strains were adjusted to 0.5 McFarland in a sterile 0.9% NaCl solution, containing approximately 10^8^ CFU·mL^−1^ for bacterial strains and 10^7^ CFU·mL^−1^ for yeast strains [15].

### 4.5. Antimicrobial Assay

The antimicrobial activity of *F. dilatata* ethanol extract was assessed using both the disk diffusion test and the MIC determination, while the water extract was evaluated solely through the MIC method due to testing limitations. The disk diffusion test for the ethanol extract followed the protocol described by Andrews [39]. Initially, Mueller Hinton Agar (BD Difco, USA) was poured into a 90 mm sterile Petri dish to a thickness of 4.0 mm ± 0.5 mm. The ethanol extract of *F. dilatata* was applied to 6 mm Oxoid antimicrobial susceptibility test disks in quantities of 50, 100, and 200 µL. The ethyl alcohol on the disks, which might affect the test results, was allowed to evaporate by drying overnight at 30 °C under sterile conditions. Subsequently, the culture media were inoculated with the previously prepared inoculum. In the final step, the extract-loaded disks were placed on the media and incubated. At the end of the incubation period, the diameters of the inhibition zones formed around the disks were measured and recorded in millimeters [15]. Gentamicin was used as a positive control, and its results are also included in the Table 1. Disks impregnated with 200 µL of absolute ethanol, without the extract, served as the negative controls. The ethanol was allowed to evaporate under the same conditions and duration as in the experimental procedure, resulting in no observed inhibition zones.

The minimum inhibitory concentration (MIC) values for the *F. dilatata* ethanol and water extracts were ascertained utilizing the broth microdilution method, following the approach presented by Kowalska–Krochmal and Dudek–Wicher [40]. Mueller–Hinton broth (MHB) was the chosen medium for the cultivation of various microbial strains. The cell density for each reference strain solution was adjusted to correspond to the 0.5 McFarland standard (1.5 × 10^8^ CFU/mL). A range of dilutions for the plant materials was prepared, and 100 µL from each dilution was pipetted into the wells of a 96-well plate. Subsequently, 50 µL of the microbial inoculum was introduced to achieve a final volume of 100 µL per well. Microbial growth was assessed by spectrophotometrically supported visual examination. The positive control comprised MHB containing the test microorganisms. The MIC corresponds to the lowest concentration of the plant sample required to inhibit bacterial growth after incubation for 24 h. The findings were presented in mg/mL, and the experiments were conducted in triplicate for accuracy.

### 4.6. Antioxidant Activity by DPPH Radical Scavenging Method

The antioxidant activity of *F. dilatata* ethanol extract was determined using the DPPH method, which is based on measuring the extract’s antioxidant capacity to scavenge DPPH radicals. The DPPH solution for the study was prepared by adding 3.9432 mg of DPPH (2,2-diphenyl-1-picrylhydrazyl) to 50 mL of ethanol and shielding it from light [41]. The DPPH solvent was added to the *F. dilatata* extract and incubated for 30 min at room temperature in a dark environment. After completing the incubation, the absorbance at 515 nm was measured using a spectrophotometer (Biotek Microplate Spectrophotometer, Winooski, VT, USA). Ascorbic acid served as a positive control for the DPPH test [42]. For the negative controls, ethanol without the extract was used.

### 4.7. Gas Chromatography-Mass Spectrometry (GC-MS) Analysis

Gas chromatography/mass spectrometry (GC/MS) is a combination of two analytical techniques: gas chromatography separates the components in a mixture, while mass spectrometry defines the components structurally [43]. In this study, an Agilent model 8890 GC/MS instrument was used. The injector temperature was set at 350 °C, and helium gas served as the carrier gas (1 mL/min). The injection mode was a 10:1 split, with an injection volume of 1 µL. The oven temperature was programmed to increase from 40 °C to 150 °C at a rate of 4 degrees per minute, from 150 °C to 180 °C at 3 degrees per minute, from 180 °C to 230 °C at 2 degrees per minute, and from 230 °C to 280 °C at 1 degree per minute [15]. The ions formed as a result of electron ionization using the GC/MS technique were separated according to their mass-to-charge ratio and recorded in the detector. The data were then collected from the computer. The compounds were identified by matching them with the data of compounds in the latest NIST and Wiley data libraries.

### 4.8. Statistical Analysis

All experiments were conducted in triplicate. A one-way ANOVA was employed for the statistical analysis, with a significance level set at 0.05.

## 5. Conclusions

This study highlights the promising antimicrobial and antioxidant potential of *F. dilatata* ethanol extract. Our results demonstrated significant antimicrobial activity against various bacterial strains, including Gram-positive and Gram-negative bacteria, as well as multidrug resistant strains. In addition, the extract exhibited moderate antioxidant activity. The GC-MS analysis revealed the presence of several bioactive compounds, which may be responsible for the observed activities. However, the possibility of undiscovered compounds and their synergistic or antagonistic effects cannot be ruled out. Future studies should focus on the identification of these compounds and their specific contributions to the extract’s bioactivity. Furthermore, investigations into the extract’s mode of action and its potential for development into novel therapeutic agents are warranted. The findings of this study contribute to the growing body of evidence supporting the potential of liverwort-derived compounds, which may pave the way for the development of synthetic production methods for these bioactive molecules in the pharmaceutical and food industries.

In addition to the promising antimicrobial and antioxidant potential of *F. dilatata* ethanol extract, the GC-MS analysis identified Frullanolide as a primary bioactive compound, which is known for its anticancer properties. This suggests that future studies may investigate the potential anticancer effects of *F. dilatata* extract, specifically focusing on Frullanolide.

## Figures and Tables

**Table 1:** Antimicrobial susceptibility assessed by disk diffusion method.

Microorganisms	50 µL	100 µL	200 µL	*p*-Value *	GEN (10 µg)
*Acinetobacter baumannii* CECT 9111	0.00 ± 0.00	0.00 ± 0.00	0.00 ± 0.00	-	13
*Acinetobacter baumannii* MDR	0.00 ± 0.00	0.00 ± 0.00	0.00 ± 0.00	-	10
*Candida albicans* DSMZ 1386	0.00 ± 0.00	0.00 ± 0.00	0.00 ± 0.00	-	12
*Candida tropicalis* CI	0.00 ± 0.00	0.00 ± 0.00	0.00 ± 0.00	-	0
*Escherichia coli* ATCC 25922	0.00 ± 0.00	0.00 ± 0.00	0.00 ± 0.00	-	22
*Enterococcus faecalis* ATCC 29212	7.00 ± 0.00	8.00 ± 0.00	9.00 ± 0.00	<2.2 × 10^−16^ **	12
*Enterococcus faecium* FI	7.00 ± 0.00	8.00 ± 0.00	8.00 ± 0.58	0.125	28
*Klebsiella pneumoniae* CI	0.00 ± 0.00	0.00 ± 0.00	0.00 ± 0.00	-	18
*Listeria monocytogenes* ATCC 7644	8.00 ± 0.00	9.00 ± 0.00	9.00 ± 0.58	0.125	28
*Pseudomonas aeruginosa* DSMZ 50071	0.00 ± 0.00	0.00 ± 0.00	0.00 ± 0.00	-	15
*Providencia rustigianii* MDR	7.00 ± 0.00	8.00 ± 0.00	9.00 ± 0.00	<2.2 × 10^−16^ **	16
*Staphylococcus aureus* ATCC 25923	9.00 ± 0.58	10.00 ± 0.00	11.00 ± 0.00	0.016	21
*Staphylococcus hominis* ATCC 27844	7.00 ± 0.00	8.00 ± 0.00	0.00 ± 0.00	<2.2 × 10^−16^ ***	18
*Streptococcus pneumoniae* MDR	0.00 ± 0.00	0.00 ± 0.00	0.00 ± 0.00	-	10
*Salmonella typhimurium* SL 1344	0.00 ± 0.00	0.00 ± 0.00	0.00 ± 0.00	-	24

Data presented as mean values ± standard errors. GEN: gentamicin FI: food isolate CI: clinical isolate MDR: multidrug resistant. An ANOVA test was performed on the three replicates to determine the significance of differences between them, yielding a *p*-value of 0.9625. * The *p*-values presented in this table represent the significance of the differences between the three dosage levels (50 µL, 100 µL, 200 µL) for each microorganism, as determined by one-way ANOVA ** F value = 1.1756 × 10^32^, ANOVA F-tests on an essentially perfect fit are unreliable, *** F value = 9.2488 × 10^32^, ANOVA F-tests on an essentially perfect fit are unreliable.

**Table 2:** Minimum inhibitory concentration (MIC) values for ethanol and water extracts.

Microorganisms	Ethanol Extract MIC (mg/mL)	Water Extract MIC (mg/mL)
*Acinetobacter baumannii* CECT 9111	-	-
*Acinetobacter baumannii* MDR	-	-
*Candida albicans* DSMZ 1386	0.257	-
*Candida tropicalis* CI	0.514	-
*Escherichia coli* ATCC 25922	-	-
*Enterococcus faecalis* ATCC 29212	0.1285	-
*Enterococcus faecium* FI	1.028	-
*Klebsiella pneumoniae* CI	-	-
*Listeria monocytogenes* ATCC 7644	0.257	21.44
*Pseudomonas aeruginosa* DSMZ 50071	-	-
*Providencia rustigianii* MDR	1.028	-
*Staphylococcus aureus* ATCC 25923	0.257	21.44
*Staphylococcus hominis* ATCC 27844	-	-
*Streptococcus pneumoniae* MDR	-	-
*Salmonella typhimurium* SL 1344	-	-

FI: food isolate CI: clinical isolate MDR: multidrug resistant -: No activity based on the three replicates conducted, and no differences were observed in the results, leading to an ANOVA value of 1.

**Table 3:** DPPH radical scavenging activity results.

Concentration (µg/mL)	*FD* (%)	AA (%)	*t*-Value	*p*-Value
200.000	89.294	94.515	−4.702	0.063
100.000	85.654	93.654	−4.728	0.063
50.000	65.456	92.878	−13.916	0.012
25.000	43.099	90.501	−11.787	0.016
12.500	35.813	68.095	−5.075	0.058
6.250	16.224	47.973	−2.321	0.144
3.125	10.436	28.418	−3.293	0.097
1.075	10.002	20.788	−1.607	0.232

*FD*: *F. dilatata* scavenging AA: ascorbic acid scavenging. The *t*-value represents the test statistic from the independent two-sample *t*-test comparing FD and AA at each concentration. The *p*-value is the probability of observing a *t*-value as extreme or more extreme than the calculated one under the null hypothesis, assuming no significant difference between the means. A smaller *p*-value (typically below 0.05) indicates a statistically significant difference. The ANOVA *p*-value between the three parallels was 0.997492, and the Pearson correlation coefficient was 0.8690298. When evaluating FD and AA across all concentrations using ANOVA, the *p*-value was 0.1754.

**Table 4:** GC-MS Analysis of *F. dilatata* Ethanol Extract Components.

No	RT	Chemical Structure	Compound Name	Formula	Molecular Weight (g/mol)	Area (%)	Known Activity
1	21.456	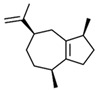	Azulene,1,2,3,4,5,6,7,8-octahydro-1,4-dimethyl-7-(1-methylethenyl)-, (1S,4S,7R)-	C_15_H_24_	204.351	2.19	-
2	22.272	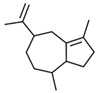	Aciphyllene	C_15_H_24_	204.351	0.77	-
3	22.398	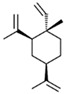	.beta.-Elemene	C_15_H_24_	204.351	0.84	Antioxidant activity [20], antitumor effect [21], antiproliferative effect [22]
5	24.620	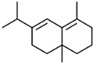	delta-Selinene	C_15_H_24_	204.35	2.19	-
6	28.048		Unknown	-	-	0.5	-
7	29.246	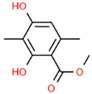	Atraric acid	C_10_H_12_O_4_	196.200	0.92	Anti-inflammatory effect [23]
8	30.240		Unknown	-	-	1.73	
9	31.547	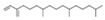	Neophytadiene	C_20_H_38_	278.516	1.18	-
10	32.321		Unknown	-	-	1.25	
11	33.000		Unknown	-	-	3.39	
12	33.157		Unknown	-	-	3.19	
13	33.634	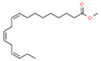	Methyl linolenate	C_19_H_32_O_2_	292.456	0.59	-
14	33.793	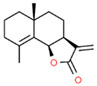	Frullanolide	C_15_H_20_O_2_	232.318	19.08	Anti-breast cancer activity [24]
15	33.921		Unknown	-	-	1.34	
16	34.068	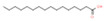	Palmitic acid	C_16_H_32_O_2_	256.424	9.83	Antitumor activity [25], antiviral activity [26]
17	34.403	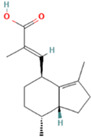	Valerenic acid	C_15_H_22_O_2_	234.334	5.3	Anxiolytic effect [27]
18	34.862	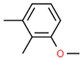	2,3-Dimethylanisole	C_9_H_12_O	136.191	15.21	-
19	36.885	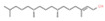	Phytol	C_20_H_40_O	296.531	1.01	Anti-quorum sensing activity [28], antimicrobial activity [29]
20	37.530	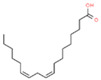	Linoleic acid	C_18_H_32_O_2_	280.445	11.11	Antioxidant activity [30]
21	41.087	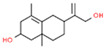	Bicyclo [4.4.0]dec-5-ene,1,5-dimethyl-3-hydroxy-8-(1-methylene-2-hydroxyethyl-1)-	C_15_H_24_O_2_	236.350	3.89	-
22	45.183		Unknown	-	-	1.66	-
23	45.866	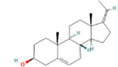	(Z)-pregna-5,17(20)-dien-3beta-ol	C_21_H_32_O	300.500	2.52	-

## Data Availability

All data that were generated or analyzed during this study have been included in this published article.
